# The efficacy of maxillary protraction protocols with the micro-implant-assisted rapid palatal expander (MARPE) and the novel N2 mini-implant—a finite element study

**DOI:** 10.1186/s40510-015-0083-z

**Published:** 2015-06-04

**Authors:** Won Moon, Kimberley W. Wu, Matthew MacGinnis, Jay Sung, Howard Chu, George Youssef, Andre Machado

**Affiliations:** UCLA Section of Orthodontics, UCLA School of Dentistry, 10833 Le Conte Avenue, CHS – Box 951668, Los Angeles, CA 90095-1668 USA; Mechanical Engineering Department, California State University, Northridge, 18111 Nordhoff Street, Northridge, CA 91330 USA; Section of Orthodontics, Federal University of Bahia, Salvador, Bahia Brazil

## Abstract

**Background:**

Maxillary protraction with the novel N2 mini-implant- and micro-implant-assisted rapid palatal expander (MARPE) can potentially provide significant skeletal effects without surgery, even in older patients where conventional facemask therapy has limited skeletal effects. However, the skeletal effects of altering the location and direction of force from mini-implant-assisted maxillary protraction have not been extensively analyzed. In this study, the application of the novel N2 mini-implant as an orthopedic anchorage device is explored in its ability to treat patients with class III malocclusions.

**Methods:**

A 3D cranial mesh model with associated sutures was developed from CT images and Mimics modeling software. Utilizing ANSYS simulation software, protraction forces were applied at different locations and directions to simulate conventional facemask therapy and seven maxillary protraction protocols utilizing the novel N2 mini-implant. Stress distribution and displacement were analyzed. Video animations and superimpositions were created.

**Results:**

By changing the vector of force and location of N2 mini-implant, the maxilla was displaced differentially. Varying degrees of forward, downward, and rotational movements were observed in each case. For brachyfacial patients, anterior micro-implant-supported protraction at −45° or intermaxillary class III elastics at −45° are recommended. For dolicofacial patients, either anterior micro-implants at −15° or an intermaxillary spring at +30° is recommended. For mesofacial patients with favorable vertical maxillary position, palatal micro-implants at −30° are recommended; anterior micro-implants at −30° are preferred for shallow bites. For patients with a severe mid-facial deficiency, intermaxillary class III elastics at −30° are most effective in promoting anterior growth of the maxilla.

**Conclusions:**

By varying the location of N2 mini-implants and vector of class III mechanics, clinicians can differentially alter the magnitude of forward, downward, and rotational movement of the maxilla. As a result, treatment protocol can be customized for each unique class III patient.

## Background

Facemask has shown some effectiveness in modifying growth and eliminating surgery in select patients [1]; however, there are many limitations. Unwanted dental side effects include proclination of maxillary incisors and extrusion and mesial tipping of the maxillary molars [2–5]. In addition, the rotation of the occlusal plane with conventional facemask has been observed leading to the development of an adjustable facemask that allows altering the line of force to achieve the desired skeletal movement [6].

To overcome these limitations, some researchers propose the use of miniplates for skeletal anchorage [7, 8], which has resulted in greater skeletal effects, even in older patients. Numerous studies have shown the effectiveness of the use of miniplates placed in different locations within the maxilla in the clinical treatment of class III patients [9, 10]. While Lee et al. [10] showed that the site of miniplate placement should be specifically considered between the infrazygomatic crest and the lateral nasal walls, Kim et al. [9] showed that the placement of miniplates in the palatal area resulted in wider stress distribution and more forward displacement compared to miniplates placed at the infrazygomatic crest area and conventional tooth-borne appliances.

With the use of micro-implants, it is now possible to achieve skeletal anchorage without the surgical procedures involved with placement and removal of miniplates. While micro-implant-supported facemask has shown evidence of clinical success [11, 12], few studies have been published on this technique and few have analyzed how micro-implants can be utilized for maximal clinical effectiveness [11, 13, 14].

With the recent development of the novel N2 mini-implant (3-mm diameter, 2-mm length, tapered shape), it is believed that the short length of the implant reduces the risk of damaging anatomic structures during placement and therefore does not need to be placed interradicularly [15–17]. The aim of this study is to examine the ability of the novel micro-implant-assisted rapid palatal expander (MARPE) and N2 mini-implant to serve as an orthopedic anchorage device in creating favorable maxillary protraction protocols in lieu of the more invasive miniplates.

A finite element approach was used to simulate conventional facemask therapy, MARPE, and seven novel N2 mini-implant-supported maxillary protraction protocols to evaluate the corresponding stress patterns and skeletal changes. Our objective was to explore the ability of the novel N2 mini-implant to be used as an orthopedic anchorage and to understand how different placement locations and force directions can be used to correct different types of class III malocclusions.

## Methods

The finite element method (FEM) model was generated from CT volumetric data (slice thickness of 0.300 mm) of a 42-year-old male patient of the Department of Biomedical Sciences at Ohio University, where informed consent was obtained prior to data collection. DICOM raw data was extracted from the CT and imported into Mimics 13.1 software (Materialise, Leuven, Belgium) to reconstruct a 3D model (Wu Laboratory, UCLA Bioengineering).

**Fig. 1:**
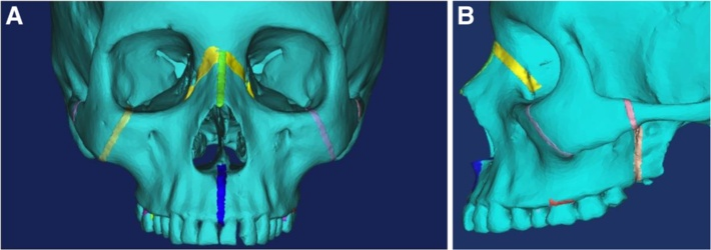
3D skull with manually generated sutures. **a** Frontal view. **b** Lateral view

Utilizing Mimics, sutures 1.5–2 mm in width were manually generated (Fig. 1): mid-palatal, pterygomaxillary (2), zygomaticomaxillary (2), zygomaticotemporal (2), median nasal, and lateral nasal (2). The FEM model generated from the ANSYS software yielded 91,933 elements and 344,451 nodes. Nodes along the foramen magnum and on the center of the forehead were constrained in all degrees of freedom, with zero displacement and zero rotation.

The 3D mesh was then imported into ANSYS 12.0 (ANSYS Inc, Canonsburg, PA) FEM software program. The FEM model was defined to be linear elastic and isotropic. The bone and tooth structures in the model were assigned properties of compact bone: Poisson’s ratio of *ν* = 0.3 and a Young’s modulus of *E* = 1.37 × 10^3^ (kg/mm^2^) [11, 18]. Sutural elements were assigned values of connective tissue: *ν* = 0.49 and *E* = 6.8 × 10^−2^ (kg/mm^2^) [11, 13].

Table 1 shows different locations and directions of force application, which simulate eight clinical protocols for maxillary protraction. The location of force delineates where the elastics of the facemask appliance pull from in simulation A and the location of micro-implant placement in simulations B–H. The direction of force (denoted by the [angle]) delineates protraction in relation to the occlusal plane. Values of 1000 g per side were applied for all simulations.

**Table 1:** Simulations of eight clinical protocols for maxillary protraction

Simulation	Clinical protocol	Location of force (bilaterally)	Direction of force (to occlusal plane)
A	FM [−30]	Buccal surface of maxillary first molars	−30
B	Pal-MI-FM [−30]	3-mm lateral of the mid-palatine suture	−30
C	Ant-MI-FM [−15]	Between roots of canine and first premolar	−15
D	Ant-MI-FM [−30]	Between roots of canine and first premolar	−30
E	Ant-MI-FM [−45]	Between roots of canine and first premolar	−45
F	Ant-MI-FM [+30]	Between roots of canine and first premolar	+30
G	Post-MI-FM [−30]	Between roots of second premolar and molar	−30
H	Post-MI-FM [−45]	Between roots of second premolar and molar	−45

**Fig. 2:**
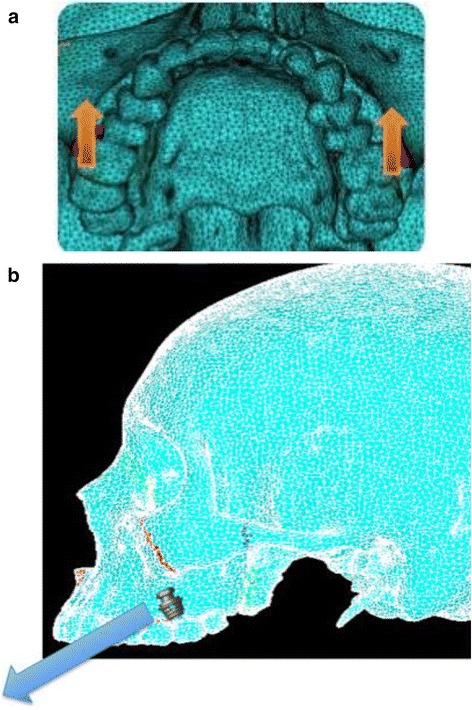
**a**, **b** Location of force application for simulation A—FM [−30°]

**Fig. 3:**
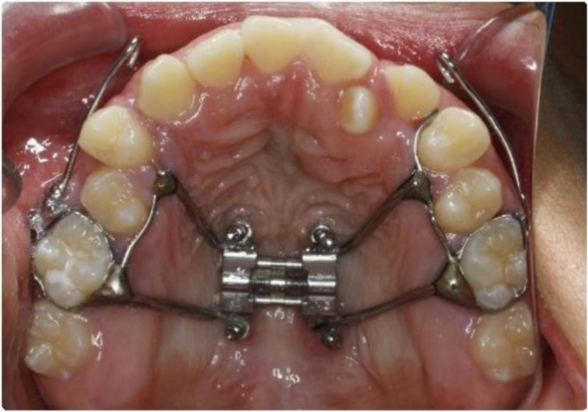
Intraoral view of micro-implant-supported hyrax with facemask with Pal-MI-FM [−30°]

**Fig. 4:**
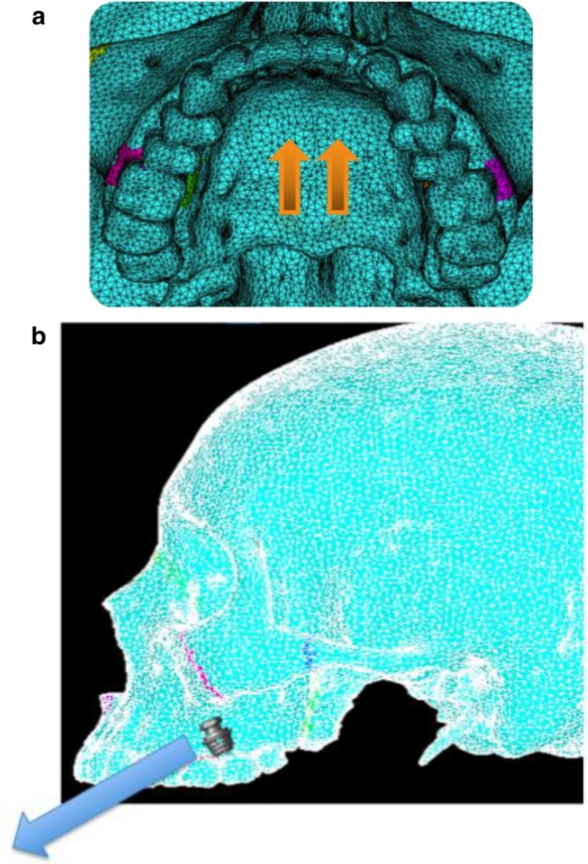
**a**, **b** Location of force application for simulation B—Pal-MI-FM [−30°]

**Fig. 5:**
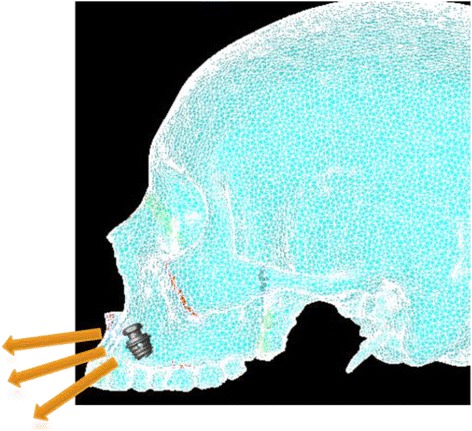
Location and direction of force application for simulation C—Ant-MI-FM[−15°], simulation D—Ant-MI-FM[−30°], and simulation E—Ant-MI-FM[−45°]

**Fig. 6:**
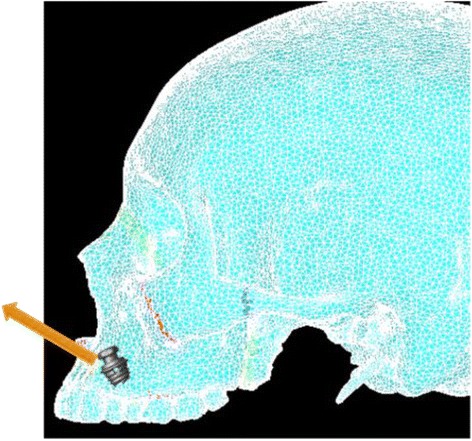
Location and direction of force application for simulation F—Ant-MI-FM [+30°]

**Fig. 7:**
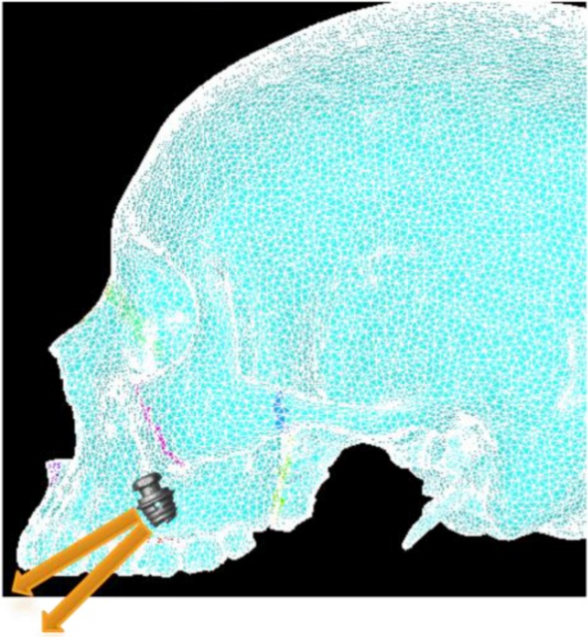
Location and direction of force application for simulation G—Post-MI-FM [−30°] and simulation H—Post-MI-FM [−45°]

Simulation A mimics conventional facemask therapy, with force applied to the buccal of the first maxillary molars, angled 30° below the occlusal plane (Fig. 2). Simulation B models a micro-implant-supported hyrax with facemask shown in Fig. 3. The forces are applied 3 mm lateral to the mid-palatal suture, at a 30° angle below the occlusal plane (Fig. 4). For simulations C, D, and E, facemask therapy directly from anterior micro-implants placed between the canine and first premolar roots is being modeled. The direction of force application is respectively 15°, 30°, and 45° below the occlusal plane (Fig. 5). Simulation F also has forces applied in the anterior between the canine and first premolar roots, but is directed 30° above the occlusal plane, simulating use of an intermaxillary spring pushing the maxilla forward and upward (Fig. 6). Finally, simulations G and H model the use of intermaxillary elastics from posterior maxillary micro-implants to anterior mandibular micro-implants. Point of force application is in between the roots of the second premolar and first molar, directed 30° and 45° below the occlusal plane (Fig. 7).

With ANSYS software:

1. Tensile and compressive stresses were measured separately in all simulations.
2. Video animations were developed.
3. Superimpositions were created to depict the skeletal displacement as a result of altering the location and direction of force application.

## Results

**Fig. 8:**
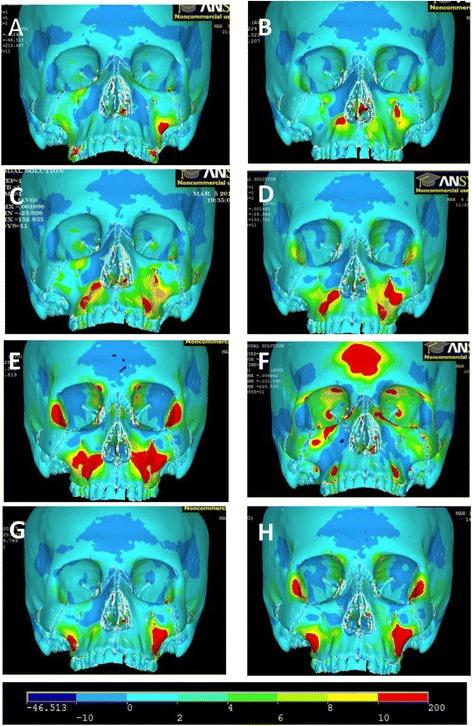
Frontal view of first principle stresses. **a** FM [−30°]. **b** Pal-MI-FM [−30°]. **c** Ant-MI-FM [−15°]. **d** Ant-MI-FM [−30°]. **e** Ant-MI-FM [−45°]. **f** Ant-MI-FM [+30°]. **g** Post-MI-FM [−30°]. **h** Post-MI-FM [−45°]

**Fig. 9:**
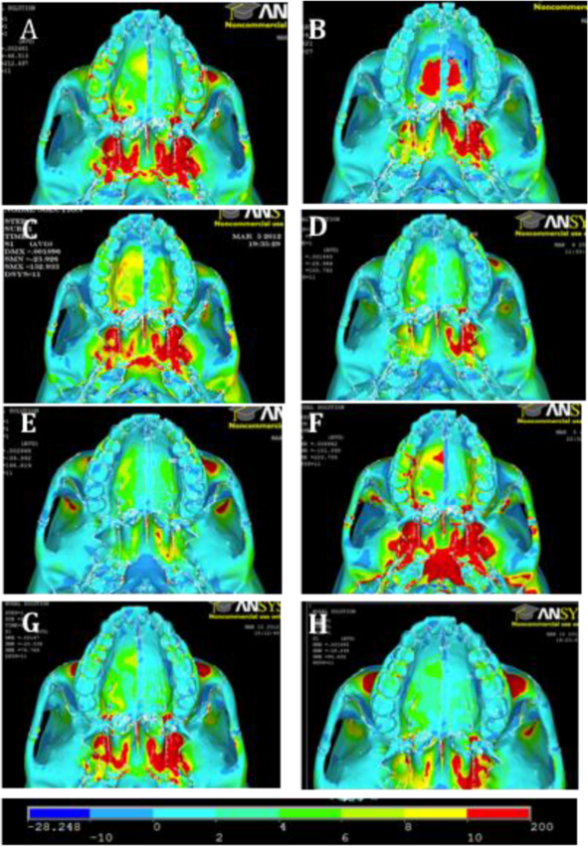
Occlusal view of first principle stresses. **a** FM [−30°]. **b** Pal-MI-FM [−30°]. **c** Ant-MI-FM [−15°]. **d** Ant-MI-FM [−30°]. **e** Ant-MI-FM [−45°]. **f** Ant-MI-FM [+30°]. **g** Post-MI-FM [−30°]. **h** Post-MI-FM [−45°]

First principle stress distributions depicting tensile stress are shown (Figs. 8 and 9). The color scale at the bottom of the figure shows the distribution of stress from the lowest (*blue*) to the highest (*red*).

**Fig. 10:**
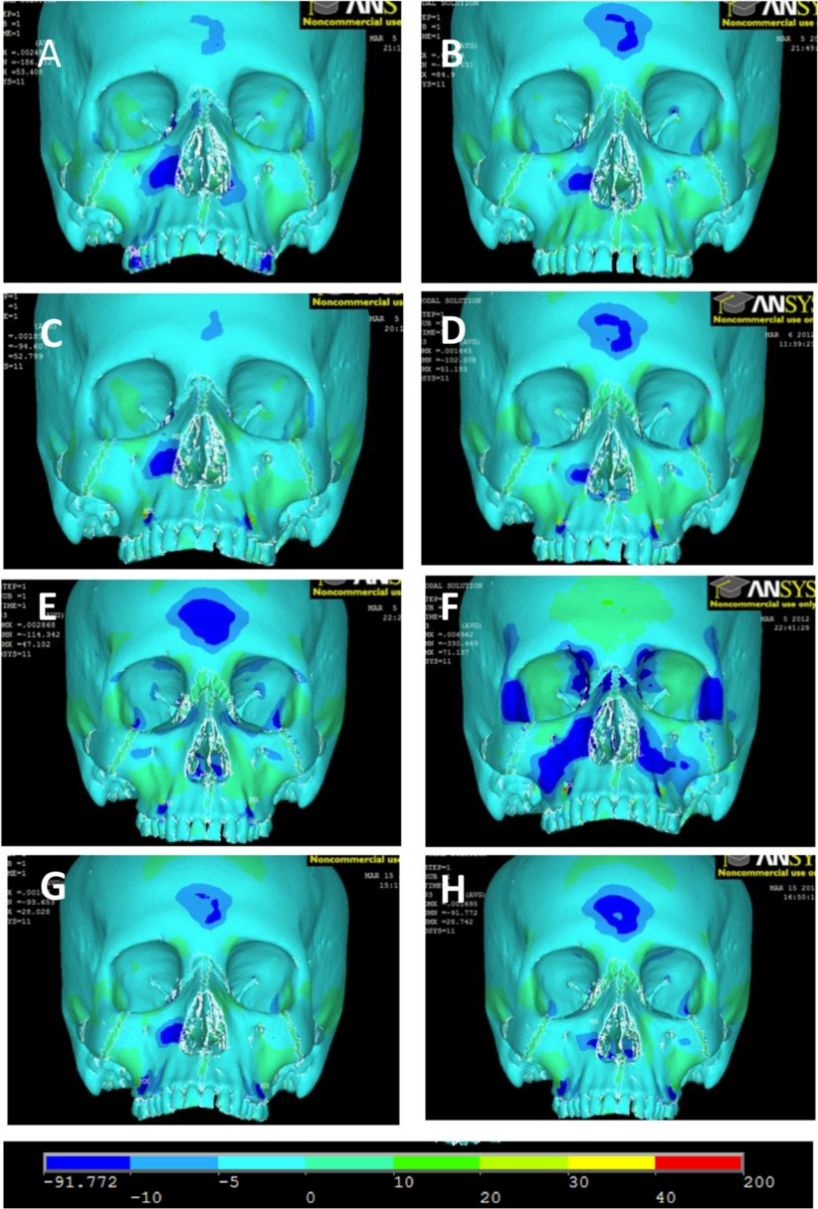
Frontal view of third principle stresses. **a** FM [−30°]. **b** Pal-MI-FM [−30°]. **c** Ant-MI-FM [−15°]. **d** Ant-MI-FM [−30°]. **e** Ant-MI-FM [−45°]. **f** Ant-MI-FM [+30°]. **g** Post-MI-FM [−30°]. **h** Post-MI-FM [−45°]

For all simulations, there is a high concentration of tensile stress directly posterior to the location of force application. In addition, tensile stresses concentrate at the pterygoid plates in all simulations, with slightly lower levels in simulation E. Tensile stresses are also evident in the medial orbit of all simulations, with greater levels in simulations E, F, and H. Other simulations have several areas of stress concentration unique to the simulation. In simulation A, tensile stresses are also present in the maxillary buttress. In simulations E and H, tensile stresses congregate near the lateral orbit, as well as the frontal process distal to the zygoma. The most unique pattern of tensile stress involves simulation F, with additional tensile stresses involving the forehead, orbit, zygomaticomaxillary suture, frontal process of the zygoma, and extending from the pterygoid plates backward to the foramen magnum.

Third principle stresses depicting compressive stress are also shown (Fig. 10). *Dark blue areas* show areas of high compressive stress.

**Fig. 11:**
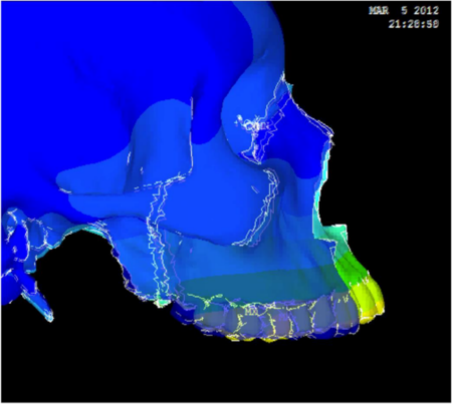
Simulation A superimposition

For all simulations, there is a high concentration of compressive stress directly anterior to the location of force application. All simulations display compressive stresses in the forehead, with significantly less in simulation F. There is also a general concentration of stresses lateral to the infraorbital foramen, although this is minimal in simulations E and H. The distribution of stresses is distinctly unique in simulation F, with areas of compressive stress involving the lateral border of the orbit, medial superior border of the orbit, maxillary buttress, zygoma, and in the frontal bone distal to the zygoma.

**Fig. 12:**
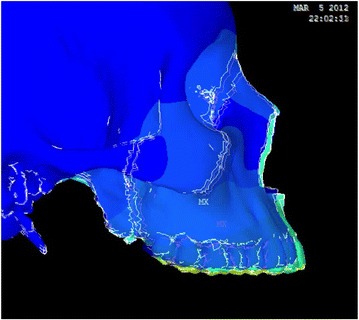
Simulation B superimposition

**Fig. 13:**
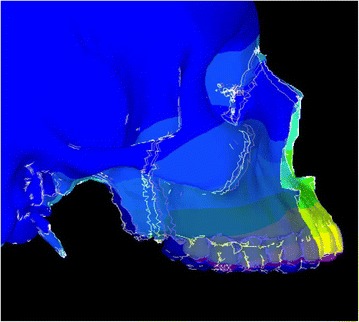
Simulation C superimposition

**Fig. 14:**
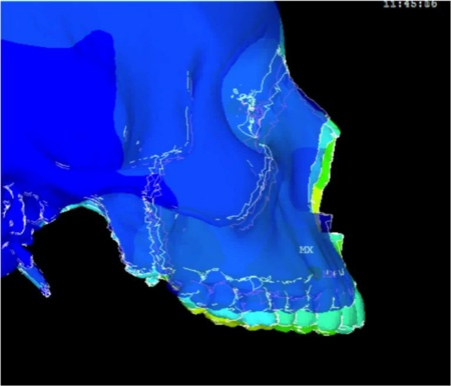
Simulation D superimposition

**Fig. 15:**
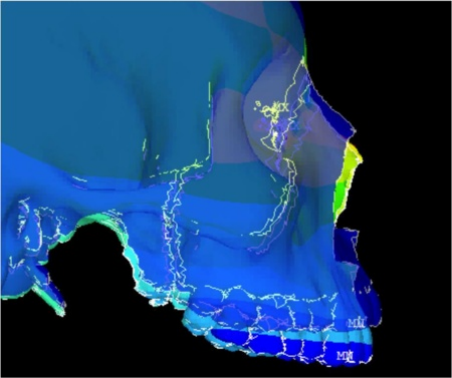
Simulation E superimposition

**Fig. 16:**
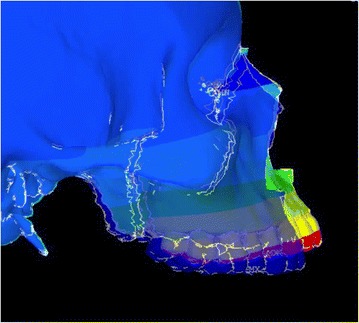
Simulation F superimposition

**Fig. 17:**
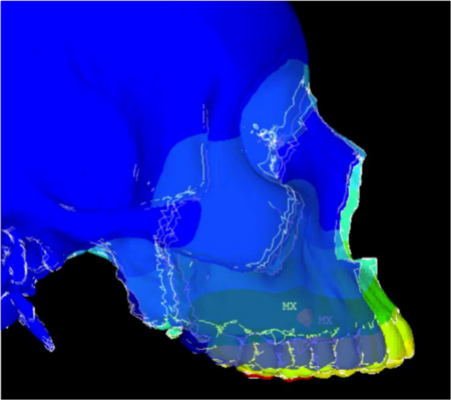
Simulation G superimposition

**Fig. 18:**
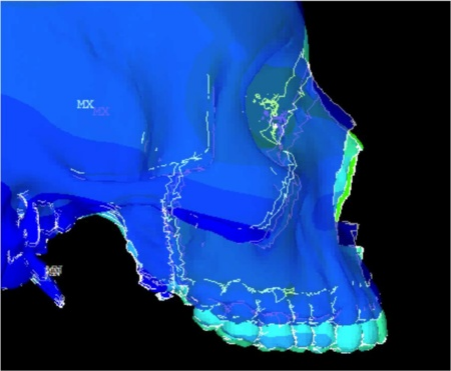
Simulation H superimposition

Animation videos and superimpositions of each simulation were created. (simulations A–H). For the superimpositions, the “before” image is shown in *blue*, while the “after” image is displayed in a *range of colors* that directly correspond to the amount of Y-displacement (pure protraction) following force application (Figs. 11, 12, 13, 14, 15, 16, 17, 18). As the color approaches *red* in the rainbow spectrum of colors, there is more Y-displacement of the skull model. Table 2 summarizes the significant findings from each simulation video and superimposition.

**Table 2:** Skeletal effects on the maxillary complex

Simulation	Clinical protocol	Movement of maxillary complex	Details of maxillary movement
A	FM [−30]	Counter-clockwise rotation	Forward and upward movement; slight posterior downward movement
B	Pal-MI-FM [−30]	Translates forward and downward	Equal forward and downward movement
C	Ant-MI-FM [−15]	Counter-clockwise rotation	Forward and upward movement; entire maxilla moves upward
D	Ant-MI-FM [−30]	Translates; slight clockwise rotation	Forward and downward movement
E	Ant-MI-FM [−45]	Clockwise rotation	Downward and backward movement; nasal bone protracted forward
F	Ant-MI-FM [+30]	Counter-clockwise rotation	Forward and upward movement; entire maxilla moves upward
G	Post-MI-FM [−30]	Translates forward	Significant forward movement; slight downward movement
H	Post-MI-FM [−45]	Clockwise rotation	Slight forward, but mainly downward movement of maxilla

## Discussion

The hypothesis being tested in this study is that different placement locations and forces applied on the novel N2 mini-implants can translate into different types of maxillary protraction. The development of different clinical protraction protocols was achieved through the various simulations.

For all simulations, 1000 g of protraction forces were applied, as studies have shown that 500–1500 g is an appropriate force load for maxillary protraction [19]. As expected, tensile stresses were visible posterior to the site and direction of force application, while compressive stresses were observed anterior. This phenomenon was observed in all simulations. This is logical, as pulling anteriorly will tend to compress the anterior and place tension on the posterior.

The video animations and superimpositions clearly portray the skeletal effects in each simulation. Because the attachment of the maxilla to the skull is complex, the center of rotation changes depending on the location and vector of force. Varying the location and direction of force produces translation, clockwise rotation, or counter-clockwise rotation of the maxillary complex. For example, a force that is located more anteriorly in the maxilla with a strong downward vector will tend to tip the anterior segment downward. The contour plots showing first and third principle stresses also correspond with the general rotation or translation of the maxilla.

However, simulations that result in the same rotation or translation movement do not necessarily have the same anterior-posterior movement, vertical movement, and rotation. A simulation may result in upward movement of the maxilla, while another results in downward movement. In addition, the amount of forward displacement also depends on the location and direction of force. By varying the location and vector of force, the effect on sutural expansion changes. The complex anatomy of the skull and circummaxillary sutures directly influences the skeletal response from different maxillary protraction protocols. As a result, the video animations and superimpositions provide valuable additional information that can be applied to treatment of patients in the clinical setting.

Several simulations display counter-clockwise rotation of the maxilla. Conventional facemask therapy has the point of force application furthest from the maxillary fulcrum of rotation. As a result, there is significant counter-clockwise rotation. The simulation is an oversimplification, as the force is applied directly to the maxillary molars, and incorporation of periodontal ligament (PDL) into the FEM model would have revealed extrusion, buccal tipping, and mesial movement of the maxillary molars and proclination of the maxillary incisors. Also, force dissipation to the PDL results in greater dentoalveolar compensations and fewer skeletal effects. PDL could not be incorporated into the FEM model, as the element size was larger than the width of PDL. Even with PDL, the simulation will not show alveolar bone remodeling as the teeth are displaced, and the dental movement will be underestimated when using the FEM. As a result, incorporation of PDL still produces an imperfect model.

Anterior-micro-implants-facemask (Ant-MI-FM) [−15°] and Ant-MI-FM [+30°] also display counter-clockwise rotation. Ant-MI-FM [−15°] symbolizes protraction directly from anterior micro-implants while Ant-MI-FM [+30°] simulates an intermaxillary spring from mandibular posterior micro-implants to anterior maxillary micro-implants. The superimposition shows that Ant-MI-FM [−15°] impacts the maxillary complex. However, it is important to note that upward and posterior displacement will restrict maxillary growth, but will not necessarily impact it, since growth cannot be reversed or undone. Ant-MI-FM [−15°] would be beneficial in patients who display a high mandibular angle and excessive incisor show, as the force vector restricts downward displacement of the anterior maxilla. Significantly, more noticeable impaction of the maxillary complex is present in Ant-MI-FM [+30°] superimposition. This clinical protocol is unique and would be beneficial for class III malocclusions with maxillary excess displaying excessive gingiva both in the anterior and posterior regions. This protocol also results in significant protraction of the maxilla. Forward and downward displacement of the maxilla will promote sutural growth, resulting in forward and downward growth of the maxilla.

On the contrary, Ant-MI-FM [−45°] and posterior-micro-implants-facemask (Post-MI-FM) [−45°] display clockwise rotation of the maxilla. Interestingly, Ant-MI-FM [−45°], which simulates facemask protraction from anterior micro-implants, displaces the maxillary complex significantly downward and backwards, which is counterintuitive, given that the goal of maxillary protraction is to move the maxilla forward. In this case, the class III will be corrected by the clockwise rotation of the mandible, which is beneficial in select class III cases. Post-MI-FM [−45°], which simulates intermaxillary class III elastics from posterior micro-implants to anterior mandibular micro-implants, moves the maxilla downward and slightly forward. Both Ant-MI-FM [−45°] and Post-MI-FM [−45°] would be beneficial for severely brachyfacial patients with a deep bite, minimal incisal show, and reasonable maxillary A-P position.

The remaining simulations all translate the maxilla, but all in a slightly different manner. In Pal-MI-FM [−30°], the maxillary complex translates downward and forward equally, translating the whole mid-facial segment forward. The clinical effect is similar to a Le Fort III advancement. This protocol would benefit for mesocephalic class III patients with mid-facial deficiency and favorable vertical positioning of the maxilla. In facemask therapy involving anterior micro-implants, Ant-MI-FM [−30°], the maxillary complex mainly translates downward and forward, with slight downward tipping of the anterior region. This could be useful in mesocephalic class III individuals with a shallow bite. Lastly, Post-MI-FM [−30°], simulating intermaxillary elastics to micro-implants, mainly translates the maxilla forward, with very slight upward tipping of the anterior region. Of all the simulations, Post-MI-FM [−30°] most closely resembles pure anterior protraction of the maxilla and would benefit class III patients with severe mid-facial deficiency. This suggests that a 30° vector for intermaxillary class III elastics between micro-implants is most effective in promoting the anterior growth of maxilla.

The clinical application of these results is evident, given that no two patients are identical. Even though our overall goal in treating class III growing individuals may be to protract the maxilla, each patient presents with different clinical findings. Patients may be brachyfacial or dolicofacial, may present with a deep bite or open bite, may have varying severities of skeletal A-P discrepancy, and may differ in the vertical positions of the maxilla resulting in varying degrees of gingival display. As a result, the location and direction of pull from micro-implants can be altered for maximal clinical effectiveness, as each simulation resulted in a different skeletal effect.

While the study simulates the skeletal response to force application, no simulation is as reliable as real life, so there are some limitations involved with the study. First, the model utilized in this study was generated from a CT scan of a 42-year-old male patient. Even though the literature states that there are no differences in using an adult model for FEM studies [20], sutural morphology and bone anatomy may change slightly throughout growth.

The material properties of bone in this study were assumed to be linear elastic, isotropic, and time independent. This is a simplification, as real bone is more anisotropic and time dependent. Bone growth cannot be simulated; we can only show how the internal strain can promote or restrict growth. In addition, no distinction was made between cancellous and cortical bone. The material properties of sutures, while based on several publications, are still an approximation and may not be 100% coincident with connective tissue. Suture morphology was estimated, and due to limitations in computer memory and model element size, PDL could not be accurately incorporated into the model. Logically, the PDL should absorb a proportion of the forces resulting in displacement of the teeth. In addition, FEM cannot simulate the resorption and deposition of bone that should occur in the dentoalveolar bone.

However, it may be difficult to create a perfect model with ideal properties. Anatomy and material properties change from one person to another, thus making the problem more complex.

Without PDL, soft tissues, and remodeling within the model, the teeth are essentially ankylosed within the dentoalveolar bone. While the simulation does not accurately predict the dental effects [21–23], it does, however, demonstrate the skeletal effects, which is of primary concern when it comes to growth modification procedures like maxillary protraction. This study is valid in comparing how location and vector of force alters the skeletal effects. As a result, the location and direction of pull can be customized for treatment of different types of class III patients in the clinical setting.

## Conclusions

Depending on the location and direction of force, the maxillary complex rotates clockwise, counter-clockwise, and/or translates anteriorly and vertically. By varying the location and vector of class III mechanics, orthodontists can differentially alter the magnitude of forward, downward, and rotational movement of the maxilla. As a result, we can customize the location of micro-implants and direction of force based on the patient’s needs.
